# A Critical Assessment of the Two Previous Public Deliberation Workshops in South Korea

**DOI:** 10.1111/bioe.70190

**Published:** 2026-09-14

**Authors:** Kyungdo Lee

**Affiliations:** ^1^ Department of Medical Humanities and Social Medicine, University of Ulsan College of Medicine Seoul South Korea

**Keywords:** bioethics, cost sharing, health policy, mini‐publics, population‐level bioethics, public deliberation

## Abstract

Mini‐publics, which constitute a microcosmic public deliberation process, are increasingly being used in addressing broad policy decisions. Following the trend of applying mini‐publics in diverse policy decisions, the Korean National Health Insurance System experimentally held 16 public deliberation workshops since 2012. This study discusses the 5th and 10th workshops among the 16 conducted, using them as a case example to evaluate the utility and informativeness of mini‐publics in health policy making. After briefly explaining the method and results of the two workshops, this study reviews the main arguments that call for the use of public deliberation in societal decisions. Based on these arguments, the study concludes that the results of the two workshops are insufficient to serve as useful information for relevant health policy making and the reasons therefor. The study posits that similar to the two workshops, mini‐publics sometimes fail to provide informative results for technically oriented questions, result in serious disagreements and fall short of achieving some deliberative ideals, such as equal participation, reasonableness, and fairness. Given that running a public deliberation process is generally cost‐consuming, despite its potential benefits, the current widespread applications of mini‐publics should be carefully examined.

## 1 Introduction

Mini‐publics, which constitute a microcosmic public deliberation process [1],^1^ are increasingly being used in addressing broad policy decisions not only within the healthcare realm, but also in domains such as climate change, biobanking, and gene editing [2, 3, 4]. Even within the healthcare realm, the main scope of mini‐publics has been expanded widely, from healthcare priority setting to issues of general health policy and planning, such as emergency department crowding and social distancing [5, 6].

This trend of applying mini‐publics across a wide range of societal issues is also prevalent in South Korea. The Korean National Health Insurance System (KNHIS), as a national single‐payer in South Korea [7], despite its renowned performance on the average healthcare quality [8], is facing a challenge in reducing the current proportion of out‐of‐pocket payments in the total healthcare spending, which is prohibitively high (i.e., over 30%), to ensure financial risk protection and guarantee equitable access to essential healthcare [9, 10]. To address this challenge, KNHIS has two options: to expand the service coverage and to reduce the cost‐sharing^2^ rates for the covered healthcare services and goods. Nonetheless, these two policy options entail further contentious questions [11]: (1) What kind of health care should be prioritized in the benefit package? (2) How much cost sharing is deemed adequate and fair? Such questions are often irresolvable even among reasonable individuals, which motivates policymakers in South Korea to come up with a procedural approach, such as mini‐publics, complementing conventional political processes [12].

As a form of mini‐publics, KNHIS has experimentally held 16 public deliberation workshops since its adoption in 2012 [13]. Although the public deliberation process has not been formally legislated yet in South Korea, its results have been shared with the decision‐making committee of KNHIS [14]. This study critically discusses the two previous public deliberation workshops (the fifth and tenth) among the 16 conducted by KNHIS, wherein the lay participants deliberated on the following insurance coverage decisions: Exempting inpatient care costs for children, expanding the benefit package to include Korean traditional herbal medicine, and their adequate levels of cost sharing. Each of the two workshops will be renamed by their main subject of deliberation: the children's inpatient cost (abbreviated as CIC) workshop and the traditional medicine (abbreviated as TM) workshop.

The study aims to theoretically address the application of public deliberation processes in health policy making using the two workshops as a case example. This discussion will specifically address three main theoretical questions. First, what is the appropriate agenda for mini‐publics? Second, how should we interpret the results of mini‐publics, particularly in cases of serious disagreements among the participants? Third, how can we ensure that the consensus among the participants follows a fair and reasonable deliberation?

It is first necessary to clarify the rationale for selecting these two workshops for the discussion, which can be delineated as follows: First, the main deliberative questions addressed both in these two workshops concern the adequate levels of cost sharing for the relevant health care involved in both technical inquiry and considerations of equity and efficiency. This question is unique in consideration of previous literature, yet holds significant importance within the policy context of South Korea, mainly because cost sharing is perhaps the only way for KNHIS to manage demands for health care. Although cost sharing appears to be a commonly adopted approach, in most high‐income countries, alternative rationing mechanisms, such as capitation systems, also exist; cost sharing is not the only option to manage healthcare demands.^3^

Second, those two workshops illustrate that the results of public deliberation processes are sometimes indefinite and should be subject to thoughtful interpretations. Similar to the outcome of the TM workshop, public deliberation processes sometimes end with unresolved disagreements. In such cases, determining the interpretation of public deliberation results and their application to relevant policy‐making processes poses significant challenges. Despite a slight majority of lay participants reaching a consensus after the deliberation, as observed in the CIC workshop, some questions were raised regarding the legitimacy and fairness of disregarding dissenting minority opinions. Furthermore, it is necessary to consider what should be constituted as the result of the public deliberation process.

In the next section, the method and results of the two previous public deliberation workshops are detailed. Subsequently, whether and how those findings should be used in pertinent policy making is addressed after reviewing the main arguments that call for the application of mini‐publics in wide policy arenas. Using the two earlier workshops as a case study, this study concludes that the application of public deliberation is not always useful, beneficial, and informative in health policy making.

## 2 Method and Results of the Two Previous Public Deliberation Workshops

### 2.1 Method

The following information was obtained from the official documents regarding the two deliberation workshops, which remain inaccessible to the public. The documents are under proprietary of KNHIS, and the author has accessed the documents solely for research purposes from KNHIS. The documents contained the summarized survey results conducted before and after the informative sessions and after the deliberation. These results are fully anonymized, which makes it impossible to conduct additional analysis. The survey predominantly comprised Likert‐scale questions, such as asking participants how much they agreed or disagreed with a certain decision, and simple choice questions, such as asking participants their preferred policy options. However, some questions were open‐ended, such as asking the underlying reasons behind participants' opinions.

KNHIS organized two public deliberation workshops, engaging 30 lay participants in a 1‐day event entailing an informative session pre‐noon and a debate session post‐noon. Each session was held approximately for 2 h. The participants were selected using stratified random sampling from approximately 2000 volunteers. These volunteers were stratified by age, sex, education level, residential area, occupation, disability, and morbidity to constitute a representative sample of the entire South Korean population. The volunteers were recruited through advertisements posted on the KNHIS website and social media accounts.

The method employed in the two workshops incorporated elements from both the Citizens' Jury and Deliberative Polling. Deliberative settings with 20–30 lay participants (such as the two workshops) are commonly found in the mini‐publics called “Citizens' Jury [14, 15].” The jurors, in general, communicate the results of the Citizens' Jury through a written statement. Nonetheless, the two workshops provided their main results as serial surveys, a form that is not generally adopted by a Citizens' Jury but by Deliberative Polling. Fishkin was the first to conduct serial surveys in a mini‐public and named them Deliberative Polling [16, 17, 18]. Deliberative Polling primarily aimed to observe how the opinions of the lay participants shift throughout the deliberation process [16]. This method is commonly used in deliberative settings with a few hundred lay participants. Therefore, the settings of the two workshops were not the usual ones employed in this method. The survey questions were written, reviewed, and authorized by KNHIS, and the survey results have mostly been shared with the KNHIS's policy‐making committee, officially called the “Health Insurance Policy Review Committee”, although the committee has no obligation to reflect them in final decision making.

The demographic information for the lay participants is summarized in Table 1.

**Table 1:** Demographics of the lay participants.

Standard	Category	Expected value^a^	Number of lay participants
Sex	Males	15	15
	Females	15	15
Age	19–39 years	12	12
	40–59 years	14	14
	60–69 years	4	4
Residential area	Greater Seoul	15	15
	Others	15	15
Occupation	Managerial jobs and professionals	4	3
	Office workers, salesperson, farmers, manual laborers, and military personnel	14	13
	Student, housekeeper, retired, and unemployed	12	14
Educational level	Without a college degree	18	16
	With a college degree	12	14
Disability	Yes	1	0
	No	29	30
Chronic disease	Yes	5	8
	No	25	22

^a^ Expected value indicates the number of people expected in the representative sample, with the size of 30, of the entire South Korean population. There are some discrepancies between the expected value and the actual number of participants that fall under each category, but no specific explanation is given in the official documents.

In the CIC workshop, three experts were invited to present short lectures to the lay participants. One of them possessed a background in health economics, one in pediatrics, and the other was a political activist who had initiated the political movement for eliminating cost sharing in KNHIS. In the TM workshop, only one expert with a doctoral degree in Korean traditional medicine was invited to present the potential benefits and problems of funding Korean traditional herbal medicine. There is no specific information regarding the contents that were delivered by each expert. One facilitator with a background in health economics and medicine led the deliberation both in the two workshops.

The main topics and questions for the two deliberation workshops are summarized in Table 2. In the CIC deliberation workshop, the lay participants mainly debated whether inpatient costs for children should be exempted completely, how much cost sharing, if any, and the exemption criteria. In the TM workshop, the lay participants mainly debated whether Korean traditional herbal medicine should be covered by the KNHIS's benefit package, the coverage limits, and the adequate cost sharing.

**Table 2:** Topics and questions for deliberation.

	Main topic	Questions for deliberation
5th deliberation workshop	Inpatient costs for children	1. Should inpatient costs for children be fully exempted? 2. What should be the criteria for full or partial exemption? 3. How much cost sharing is adequate?
10th deliberation workshop	Korean traditional herbal medicine	1. Should Korean traditional herbal medicine be covered by the benefit package of KNHIS? 2. What should be the limit for the coverage of Korean traditional herbal medicine? 3. How much cost sharing is adequate?

### 2.2 Results

#### 2.2.1 The CIC Deliberation Workshop

The main survey results of the CIC deliberation workshop are presented in Figures 1, 2, 3. At the baseline, approximately 50% of the lay participants opposed cost sharing for inpatient care for children, while approximately one‐third of them remained uncertain about their opinion (Figure 1). After the informative session, approximately half of them supported cost sharing for children's inpatient costs, but the other half disagreed. After the deliberation, over 60% of the lay participants supported cost sharing, and only a quarter disagreed. Some of the main reasons for their support of cost sharing were that cost sharing (1) can prevent unnecessary inpatient care, (2) is necessary to assign a fair amount of responsibility to healthcare users, and (3) must be exempted only for patients with severe medical conditions and for the disadvantaged. Contrarily, some of the main reasons opposing cost sharing were (1) cost sharing imposes a financial burden on the economically disadvantaged, (2) reducing cost sharing for children's inpatient care is a potential solution to low birth rates, and (3) providing benefits to children will result in substantial returns to other generations, for example, it enhances the labor force. Some participants found it challenging to uphold one view over the other because (1) deciding how much inpatient care costs for children should be shared with its users depends on their financial situation and the ability to pay, (2) it is extremely complex for the public to judge whether a full exemption is warranted, even though the reasons for and against it are worth considering.

**Figure 1:**
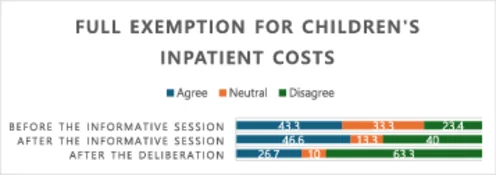
Lay participants' opinions on full exemption for children's inpatient costs, before and after the informative session and the deliberation (CIC deliberation workshop).

**Figure 2:**
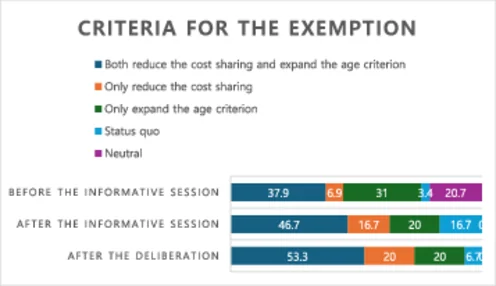
Lay participants' opinions on the criteria for exempting children's inpatient costs, before and after the informative session and the deliberation (CIC deliberation workshop).

**Figure 3:**
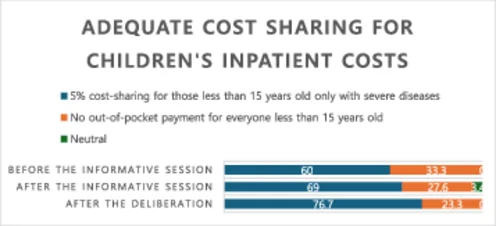
Lay participants' opinions on adequate cost sharing for children's inpatient costs, before and after the informative session and the deliberation (CIC deliberation workshop).

Regarding the second question, before the informative session, only one‐third of the participants agreed to both reducing the current coinsurance rate for children's inpatient costs from 10% to 5% and extending the age criterion for the 5% reduction in cost sharing from ages 6 to 15 (Figure 2). However, after the deliberation, approximately half of the participants agreed to the reduction of the coinsurance and expanding the age criterion. The reasons behind the change in their opinions were not documented.

In relation to the third question, prior to the informative session, 60% of the participants agreed to set the limit based on disease severity to exempt inpatient costs for children only with certain serious diseases, while imposing a 5% cost‐sharing on others. Following the deliberation, the proportion of participants supporting the opinion went up to 77% (Figure 3). The reasons for the change in their opinions were not documented.

#### 2.2.2 The TM Deliberation Workshop

The results of the TM deliberation workshop are presented in Figures 4, 5, 6. Participants' opinions remained the same throughout the workshop regarding the first question about their assent to include Korean traditional herbal medicine in the KNHIS's benefit package (Figure 4). Following the deliberation, approximately 55% of the lay participants supported the insurance coverage of Korean traditional herbal medicine, whereas 31% did not favor the decision. The main reasons for their agreement were that Korean herbal medicine (1) is often expensive, (2) is prescribed only by established medical professionals with Korean medical degrees, and (3) is used by many Koreans. In contrast, the main reasons against its coverage were: (1) it would create a huge negative budgetary impact, (2) only a limited segment of the Korean population would use it, and (3) its safety and effectiveness remain uncertain.

**Figure 4:**
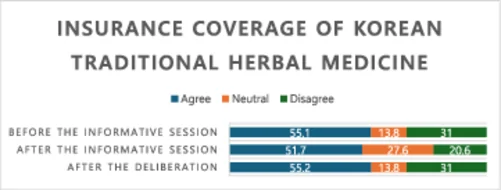
Lay participants' opinions on insurance coverage of Korean traditional herbal medicine, before and after the informative session and the deliberation (TM deliberation workshop).

**Figure 5:**
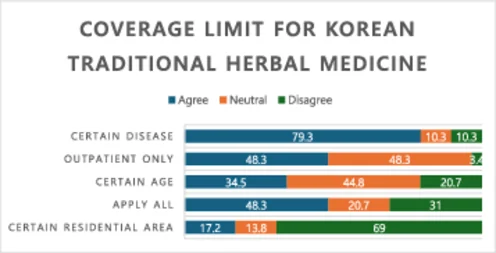
Lay participants' opinions on the coverage limit for Korean traditional herbal medicine (TM deliberation workshop).

**Figure 6:**
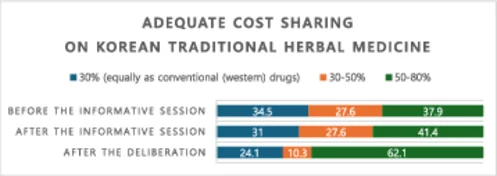
Lay participants' opinions on adequate cost sharing for Korean traditional herbal medicine, before and after the informative session and the deliberation (TM deliberation workshop).

On the second question regarding coverage limits, a survey was conducted only at the end of the deliberation workshop. Approximately 80% of lay participants agreed to limit the insurance coverage of Korean traditional herbal medicine to certain disease groups, while approximately a third agreed for certain age groups (Figure 5).

In reference to the third question about the adequate rate of cost sharing for Korean traditional herbal medicine, before the informative session, one‐third of the lay participants agreed to impose 30% cost sharing on its users equally as on the users of conventional (Western) drugs (Figure 6). After the deliberation, over half of them voted for imposing 50%–80% of the costs on its users; that is, they voted for higher cost‐sharing rates on Korean traditional herbal medicine than on conventional drugs. Some of the main reasons for this shift in their opinions were (1) the data regarding the cost‐effectiveness of traditional medicine remain indefinite, (2) it usually treats less severe health conditions compared with Western medicine, and (3) it has a negative budgetary impact.

## 3 Discussion

### 3.1 Agenda

Before addressing the question of what type of agenda is appropriate for mini‐publics, the primary use of mini‐publics must be clarified. The question primarily concerns the establishment of the right boundaries for mini‐publics, which is contingent on their primary purpose. Are mini‐publics used to make specific policy decisions, or do they merely provide information about public perceptions of underlying value conflicts? According to Fishkin, it is somewhat natural to designate some authority to mini‐publics even on specific policy matters because deliberation should be not only *for* the people but also *by* the people [17]. Fishkin posits that mini‐publics result in a prescriptive force by representing the people's true and deliberate opinions [19].

For discussion purposes, let us temporarily assume that mini‐publics are highly informative and effective in enhancing the quality, fairness, and legitimacy of final policy decisions, at least in some cases where such decisions entail serious value conflicts. Let us return to the two public deliberation workshops and, in particular, consider the deliberation question addressing how much cost‐sharing rate is adequate for Korean traditional medicine and children's inpatient costs. Are mini‐publics adequate, informative, and effective in addressing this kind of question?

Let us first focus on the CIC workshop in which, after the deliberation, over 60% of the lay participants approved the 5% cost‐sharing rate on children's inpatient care costs. One can claim that this result is informative because it shows a consensus among the majority participants that the value of efficiency should be upheld by preventing unnecessary children's inpatient care even when it might partly sacrifice the value of equity by threatening its affordability. It may seem meaningful to let the lay participants pinpoint the adequate cost‐sharing rate for children's inpatient care in order to see their priority between the conflicting values relevant to that question.

I do not intend to reject this claim fully, and perhaps it may not be entirely meaningless to let the lay participants deliberate on that question. Nonetheless, I think that it is too quick to accept that claim for several reasons. First, the way that the third deliberation question is framed seems too technical to address relevant value‐laden questions explicitly. The lay participants deliberated on which of certain limited options for cost‐sharing rate for children's inpatient care they think is adequate, not on which of relevant values should be prioritized when setting its cost‐sharing rates. Their support for the imposition of 5% cost sharing does not necessarily imply that the value of equity can be sacrificed to uphold the value of efficiency; one of their reasonings supporting the imposition of 5% cost sharing instead of 0%, for instance, was that the disadvantaged population should be excluded to justify its imposition.

Second, the information provided to the participants of the CIC workshop should have included a wide range of empirical data in order for the participants to deliberate on relevant value‐laden questions. The deliberation question, mainly due to its framing, is largely an empirical one, in a sense that addressing it needs to understand reasonable predictions of how patients' healthcare utilization decisions would be affected by adopting different cost‐sharing rates. I think that the public deliberation should have been based on the potential scenarios in which patients would make their utilization decisions under various cost‐sharing schemes, including 0%, 5%, and 10% cost sharing. Due to limited access to the materials used in the CIC workshop, there is no evidence confirming that the lay participants actually lacked those scenarios. I strongly suspect that they did, though: none of the reasoning or opinions that the lay participants provided in the survey results mentioned how patients would make their healthcare utilization decisions under different cost‐sharing schemes.

Third, even if such information had been given to the participants, it would have been too demanding and complex for them to understand reasonable predictions of the potential behavioral consequences of imposing different cost‐sharing rates on children's inpatient care. Such prediction requires a broad range of knowledge, including complex South‐Korean policy contexts, and a certain level of expertise in diverse relevant fields, such as behavioral science and economics [20, 21, 22, 23, 24]. That demandingness in required knowledge, expertise, and complexity is also cited by the lay participants in the CIC workshop as a major problem in reaching certain decisions after the deliberation: the participants who decided not to choose any certain cost‐sharing rate thought that the choice should depend on each healthcare user's financial situation and the ability to pay and that the nature of that question is extremely complex for lay publics. These reasons are worth considering. For instance, the imposition of 5% cost sharing on children's inpatient care would differentially impact their utilization decisions depending on a wide range of factors such as their socioeconomic status, their perceived necessity, and having a complementary health insurance that reimburses their out‐of‐pocket payments. In addition, even imposing 10% cost sharing does not always suffice to prevent overutilization, say, when physicians are incentivized to generate unnecessary demands.

Let us then shift to the TM workshop: the lay participants were given the task of finding the adequate cost‐sharing rate for traditional herbal medicine. The result seems somewhat informative: throughout the TM workshop, a majority of the participants became more convinced by imposing a higher cost‐sharing rate, 50%–80%, on traditional herbal medicine than on conventional western drugs. This result may be interpreted as indicating that the lay participants found it less warranted to cover traditional herbal medicine than conventional western drugs. One may find the result of the TM workshop a better example than that of the CIC workshop, in a sense that the agenda is not to pinpoint the right cost‐sharing rate for traditional herbal medicine but to comparatively decide its priority.

Still, I think that the question can be better framed to let the participants effectively deliberate on relevant value conflicts. If the intention was to find their views on the priority of the insurance coverage of traditional herbal medicine compared to that of western drugs, then why not letting them deliberate directly on the question of priority setting, instead of letting them pinpoint certain cost‐sharing rates? Again, my argument is not that all the results in the two workshops are not informative at all; some of them might imply meaningful information. Rather, my argument is that not all policy questions are adequate for public deliberation and therefore that how to frame them suitable for public deliberation is often delicate and complex. When the question for deliberation is framed in a way that does not effectively promote deliberation on relevant values, as in the two workshops, I think that the results should be subject to careful interpretations, instead of simply reflecting them in policy making.

Furthermore, if the result can be seen as implying the lay publics' comparatively low priority of covering traditional herbal medicine by national health insurance, it becomes confusing how to interpret another result of the TM workshop: at the end of the TM workshop, a slight majority of the lay participants supported the insurance coverage of traditional herbal medicine, although over 30% of them remained against it. Can this result be interpreted as implying the wide lay publics' support for the insurance coverage of traditional herbal medicine? Is that coherent with the result that the majority participants found its coverage less warranted than that of conventional drugs? In the next section, I will address such question, the problem of interpreting the results of the two workshops.

### 3.2 Interpretation of the Results

What constitutes the result of mini‐publics? The answer would partly depend on the methods of mini‐publics. The result of Deliberative Polling, for instance, is generally communicated as polling results. These polling results sometimes may be accompanied by the primary reasons underlying participants' opinions and their changes, as described in the results section of this article. Contrarily, in Citizens' Jury [14, 15], the jurors themselves usually communicate the results of the Citizens' Jury through a written statement. That type of statement generally contains not only conclusions endorsed by the majority of jurors but also, if any, remaining disagreements or minority dissenting opinions.

Irrespective of the form the results of mini‐publics can take, interpreting them is challenging, particularly when they do not end up with unanimous agreements or consensuses among the overwhelming majority. For instance, in the TM workshop, only a slight majority upheld the coverage decision of Korean traditional herbal medicine even after full deliberation. Can it be interpreted as showing the wide lay public's support for that coverage decision? Additionally, consider the CIC workshop, where slightly over 60% of the participants finally opposed the full exemption for children's inpatient costs, whereas one‐third of the participants remained in favor of this policy. Does the result of the CIC workshop imply that there was a meaningful agreement among the lay participants on that policy, or, contrarily, that there remain significant disagreements among them?

At this point, a fundamental question arises regarding the effectiveness of public deliberation in resolving remaining disagreements. According to Daniels, who in my opinion is one of the optimists who believe in the cooperative role of public deliberation, people participating in a deliberation process should not be the ones who merely seek their self‐interests but those who “seek to cooperate [with] others on terms that they can justify to each other [25].” Daniels asserts that this condition is for “narrowing down the scope of disagreement and providing the grounds on which disputes can be adjudicated [25].” Nonetheless, even if public deliberation might provide some opportunities for seeking overlapping consensus among the participants, it appears to be an empirical question as to whether and to what extent the lay participants strive to be fair‐minded in the deliberation process and succeed in reaching any meaningful consensus. This outcome would depend on numerous contextual and even arbitrary factors; therefore, while we can try to build cooperative environments in public deliberation processes, some may ultimately result in serious conflicts.

Undoubtedly, public deliberation is effective not only in identifying overlapping agreements but also in revealing serious disagreements among the public [26, 27, 28, 29, 30]. Moreover, mini‐publics may create venues in which the underlying reasons behind potential disagreements regarding certain societal matters can be fully articulated [29, 31, 32]. Despite persistent disagreements following the TM workshop, for instance, the findings can provide valuable insights for policymakers by elucidating the underlying reasons behind the disagreements around the coverage decision.

Yet, it is still uncertain how to reflect such disagreements in relevant policy making processes. Recall the result of the TM workshop, specifically the reasons why the lay participants came to their own judgments on whether to include traditional herbal medicine in the benefit package of KNHIS. The supporters' main reasonings are citing its frequent usage among the Korean population and the formal establishment of Korean traditional medicine, including its doctoral educational system separated from the one that trains “western‐style” medical doctors. The opponents' main reasonings are citing its unclear scientific evidence of effectiveness and safety, and the fact that its cultural affinity only belongs to old generations, not younger ones. How can policy makers come to a decision through reconciling this conflict in a fair manner? It may seem clear that policy makers should strive to build the scientific evidence of traditional herbal medicine. Nonetheless, building that evidence would take considerable costs and time, which could be seen as redundant by some of its users, mostly older population who strongly believe in its effectiveness and safety.

My intention is not to suggest a way to adjudicate competing claims around the insurance coverage of traditional herbal medicine. Rather, my intention is to tackle the optimism that public deliberation always results in a straightforward conclusion that policy makers can apply without further interpretation. Such interpretation entails contentious judgments particularly when the results of public deliberation merely reflect deeply entrenched value‐laden conflicts, as in the two workshops. In such cases, its application requires some additional justification, partly because it is likely to provide conflicting views and opinions only, albeit well‐considered ones.

Perhaps one result that has been overlooked is a notable shift in the participants' opinions around the insurance coverage of children's inpatient costs in the CIC workshop. Assuming that this opinion shift implies some level of consensus that was reached among the participants, is it justifiable to reflect this directly in shaping the insurance policy? I continue to remain negative, mainly because it is questionable, or at least requires more evidence, to believe that the result can be derived from a fair and reasonable deliberation. This kind of question will be addressed in the next subsection.

### 3.3 Deliberative Ideals

Given that many deliberative ideals, such as equal participation, reasonableness, and fairness, are inevitably infringed when the deliberation occurs at the macro level, a usual public deliberation process, such as mini‐publics, takes the form of microcosmic deliberation involving a few dozen lay participants. This naturally raises the issue of representation, as, despite some recruitment efforts for obtaining a representative sample of the general public, these efforts often fall short of representing diverse opinions and wills of the wider public at best. Cristina Lafont claims that compared to macro‐participation processes, micro‐deliberation would inevitably infringe on the public's equal participation in decisional processes [32, 33]. Additionally, Lafont posits that, as the lay participants of mini‐publics become better informed and are reasoned about the issues in question, their opinions, views, and values become similar to those of experts, not the general public. In other words, micro‐deliberation falls short of reflecting the public's will *as it is* [32, 33].

Many deliberative democrats, nevertheless, defend the use of mini‐publics, even when it may infringe on the equal participation of the wider public. This is mainly because some policy decisions (e.g., policies for criminal sentencing and healthcare priority setting) are often exposed to external manipulation and polarization efforts (e.g., manipulation by political elites and those with vested interests) [34]. In this regard, mini‐publics appear to create a safe place for the lay public to reasonably and fairly deliberate on certain matters, refraining from unjust external interferences. Therefore, their legitimating potential rests mostly on their distinctive capacity to achieve deliberative ideals.

Let us return to the notable shift in the opinions of the lay participants of the CIC workshop leaning toward the opposition against the full exemption policy: the proportion of those opposing the full exemption policy increased nearly three times throughout the workshop, from 23% to 63%. For discussion purposes, let us temporarily assume that this opinion change illustrates something important for policy making, say, a consensus that the arguments for the full exemption were regarded as unconvincing by the lay public. Then it seems worth asking whether that consensus is a consequence of a fair and reasonable deliberation. If it resulted from unfair, imbalanced, and unreasonable deliberation, its legitimating potential would largely be damaged.

I admit that some additional information and comprehensive analysis, for example, qualitative analysis^4^ using interviews with the invited experts and some of the lay participants, would be helpful to better assess whether that consensus came from a fair, balanced, and reasonable deliberation. This study does not conduct such analysis and such information is unavailable. Rather, my intention is mainly to suggest some possibilities that the consensus came from unfair and unreasonable deliberation, at least in some important respects. Mainly due to the lack of information that enables us to analyze the public deliberation process itself, those possibilities are speculated indirectly by analyzing the final products of public deliberation, for example, the lay participants' reasoning and conclusion.

Let us then return to the opinion change that happened in the CIC workshop and examine the main reasonings behind the opinion change. The lay participants provided their main grounds for the opinion change, and most notably, one of them was based on the expectation that the full cost exemption would induce unnecessary use of inpatient care. Another reason they provided was that 5% cost sharing is a fair share of inpatient care costs. Their reasons may seem to make some sense, nonetheless, require additional scrutiny. How can we be sure that the imposition of 5% cost sharing prevents unnecessary inpatient care while not infringing necessary use? What kind of policy options in addition to 5% cost sharing should be implemented to protect fair access to necessary inpatient care? How much would be the average 5% of children's inpatient care costs and would that be considered fair to anyone? Again, there is no available information as to whether the lay participants actually addressed such question during the deliberation process.

At least without such information, I find it reasonable to doubt that their final support for the imposition of 5% cost sharing is a conclusion that was reached through a fair and reasonable deliberation. Based on the lay participants' reasoning, for instance, some of them thought that some rationing mechanism, in this case cost sharing, should be put in place whatsoever. Then a next logical step would be for them to navigate the pros and cons of various rationing mechanisms and to deliberate on whether cost sharing would be preferable to other kinds of rationing mechanisms. Disappointingly, there is no evidence that they deliberated on that, and moreover, given that such issue is never alluded to in any of their reasoning, it is unclear, and perhaps even doubtful that they deliberated carefully enough. Because cost sharing is not the only rationing mechanism, deliberating on which of rationing mechanism is preferable in South‐Korean contexts is one of the most important value‐laden questions that need to be addressed when deciding whether to impose cost sharing for children's inpatient care. Patients in the U.K., Canada, and Scandinavian countries, for example, generally bear no cost for using essential health care at the point of service, based on the value of equity and solidarity [35]. These countries have implemented rationing mechanisms other than cost sharing, such as capitation‐based payment systems, time‐cost ordeals, and/or gate‐keeping functions of medical professionals. For instance, patients in these countries who want to visit specialists for minor health issues have to obtain a referral from their registered general practitioner, and even if they succeed in obtaining one, they have to generally wait longer than those who seek specialist care in South Korea [36].

In addition, recall that one of the lay participants' reasons for supporting 5% cost sharing was that it is a way of imposing a fair share of individual responsibility. I think that this reasoning, again, needs to be subject to additional deliberation. For instance, given that another reason for their support was that the disadvantaged population can be exempted from cost sharing, some of the supporters might have thought that cost sharing would or should be imposed only on those beyond the subsistence level. Or perhaps some others might have thought that 5% cost sharing should be imposed across the whole population including those financially disadvantaged, because they think it represents a fair amount of individual responsibility for anyone using health care by public funds. Again, there is no evidence that the lay participants went through such questions seriously, which I think are crucial in formulating specific policy options.

One may complain, rightfully so, that the entire process of the two public deliberation workshops could have been made better, perhaps to the point that my criticisms may have been inadequate. In particular, the time allotted for deliberation in the two workshops was approximately less than 4 h, which is too short to observe substantive changes in the participants' opinions and end with “considered judgments.” In addition, the facilitator and invited experts could have better assisted the deliberation process, such as posing the questions raised above and suggesting logical steps to follow.

I concede that several robust measures might have been effective in improving the overall quality of deliberation. Nonetheless, I think there still remain somewhat intricate issues that arise in actual public deliberation processes. One of them is how to ensure the equality and fairness of actual participation in a deliberation process. Recall that the majority ended up supporting the imposition of 5% cost sharing on children's inpatient care. Does that result represent the consensus that was made through deliberation among equals? How can we be sure that, during the deliberation process, the potential opinions of those extremely poor, for whom even a 5% cost sharing is unaffordable, have been reflected fairly? What I'm pointing out here is not just how to select a representative sample of lay participants with respect to their income levels, but also how to make sure that the selected participants, despite their demographical representativeness, speak up and participate equally in the actual deliberation process. The opinions of the financially disadvantaged might have been ignored or silenced because it may look irresponsible or shameful to convey that even a small amount of cost sharing can be unaffordable for themselves or others, although, again, there is no evidence that can prove such ignorance and silence actually happened in that room. At the very least, it seems overly optimistic, I think, to expect that existing biases and power imbalances, some of which are often hidden, intangible, and deeply underlying our common social practices such as communication and language, would not have influenced the deliberation in the CIC workshop [37].

Surely, every deliberation that happens in a human society may not reach the bar where such criticism does not apply. I do not intend to argue that we should abandon every deliberation process for that reason. Rather, my argument is that, provided that the legitimating force of public deliberation can be predominantly attributed to its capacity to achieve deliberative ideals, such as equal participation, reasonableness, and fairness in deliberation [37], each public deliberation process should be assessed carefully upon whether and to what extent those ideals were actually achieved. Such assessment should be taken into account when applying its results in policy making processes; if the assessment turns out to be negative in some important regards, the policy application of public deliberation needs to be indirect and nuanced rather than direct. As I pointed out above, disappointingly, the achievements of deliberative ideals would generally demand intricate efforts; their achievements hinge not just on the statistical representativeness of the lay participants, their formal education and income level, and a well‐designed informatory session, but also on the lay participants' genuine comprehension of relevant facts, their capabilities to exercise good deliberation, the capacities of facilitator, non‐verbal communications, atmosphere, and so forth.

## 4 Conclusion

Mini‐publics may work as platforms for the lay public to provide their own judgments on policy problems. Nevertheless, there remain challenges in their agenda setting, interpretation of the results, and final policy application. As in the case of the two public deliberation workshops held in South Korea, mini‐publics require tremendous efforts to provide informative results, end up with a meaningful consensus among the participants, and conduct their equal, fair, and reasonable deliberation. Accordingly, mini‐publics are not always worth applying, but only in a carefully designed and well conducted form with properly framed policy questions.

## Funding

 The author has nothing to report.

## Conflicts of Interest

The author declares no conflicts of interest.

## Data Availability

Research data are not shared.

## Endnotes

1 In this article, the term “public deliberation” is used interchangeably with “mini‐publics” as the former commonly refers to a formal process of microcosmic deliberation among the lay participants of mini‐publics.

2 Cost sharing refers to any financing arrangement where the user partly supports the cost of the services. Out‐of‐pocket payments include not only the amount of cost sharing but also the health care payments excluded in the insurance benefit package. Corina C. Ros, Peter P. Groenewegen, and Diana M. J. Delnoij, “All rights reserved, or can we just copy? Cost sharing arrangements and characteristics of health care systems,” *Health Policy* 52, no. 1 (2000/05/01/2000), https://doi.org/10.1016/S0168-8510(00)00065-8, https://www.sciencedirect.com/science/article/pii/S0168851000000658.

3 The imposition of any form of cost sharing on healthcare utilization is common. The users of some type of health care, such as primary care visits, are usually subjected to a certain level of cost sharing. This is prevalent not only in the U.S. but also in some European countries with universal health coverage like France. Patryk Perkowski and Leonard Rodberg, “Cost Sharing, Health Care Expenditures, and Utilization,” *International Journal of Health Services* 46, no. 1 (2016), https://doi.org/10.1177/0020731415615312; Ros, Groenewegen, and Delnoij, “All rights reserved, or can we just copy? Cost sharing arrangements and characteristics of health care systems.”

4 Qualitative analysis usually uses the transcript of public deliberation workshops, nonetheless because the transcript of the fifth workshop is unavailable, such analysis cannot be conducted.

## References

[1] R. E. Goodin and J. S. Dryzek , “Deliberative Impacts: The Macro‐Political Uptake of Mini‐Publics,” Politics & Society 34, no. 2 (2006): 219–244, 10.1177/0032329206288152.

[2] G. Blue , “Framing Climate Change for Public Deliberation: What Role for Interpretive Social Sciences and Humanities?,” Journal of Environmental Policy & Planning 18, no. 1 (2016): 67–84.

[3] M. M. Burgess , “From ‘Trust Us’ to Participatory Governance: Deliberative Publics and Science Policy,” Public Understanding of Science 23, no. 1 (2014): 48–52, 10.1177/0963662512472160. 24434712

[4] K. C. O'Doherty , A. K. Hawkins , and M. M. Burgess , “Involving Citizens in the Ethics of Biobank Research: Informing Institutional Policy Through Structured Public Deliberation,” Social Science & Medicine (1982) 75, no. 9 (2012): 1604–1611, 10.1016/j.socscimed.2012.06.026. 22867865

[5] N. M. Baum , P. D. Jacobson , and S. D. Goold , “Listen to the People”: Public Deliberation About Social Distancing Measures in a Pandemic,” American Journal of Bioethics 9, no. 11 (2009): 4–14, 10.1080/15265160903197531. 19882444

[6] P. A. Scuffham , N. Moretto , R. Krinks , et al., “Engaging the Public in Healthcare Decision‐Making: Results From a Citizens' Jury on Emergency Care Services,” Emergency Medicine Journal 33, no. 11 (2016): 782–788, 10.1136/emermed-2015-205663. 27323789

[7] S. Kwon , “Healthcare Financing Reform and the New Single Payer System in the Republic of Korea: Social Solidarity or Efficiency?,” International Social Security Review 56, no. 1 (2003): 75–94, 10.1111/1468-246X.00150.

[8] The Organisation for Economic Cooperation and Development , *OECD Reviews of Health Care Quality: Korea 2012* (OCED, 2012).

[9] J. Heo , J. Oh , J. Kim , et al., “Poverty in the Midst of Plenty: Unmet Needs and Distribution of Health Care Resources in South Korea,” PLoS One 7, no. 11 (2012): e51004, 10.1371/journal.pone.0051004. PMC3511433 23226447

[10] Korean Ministry of Health and Welfare , “National Health Insurance Financing and Coverage Rates,” Website National Health Insurance Financing and Coverage Rates, https://www.index.go.kr/potal/main/EachDtlPageDetail.do?idx_cd=2763.

[11] World Health Organization , Making Fair Choices on the Path to Universal Health Coverage: Final Report of the Who Consultative Group on Equity and Universal Health Coverage (World Health Organization, 2014). 10.2471/BLT.14.139139 PMC4047814 24940009

[12] N. Daniels and J. Sabin , “Limits to Health Care: Fair Procedures, Democratic Deliberation, and the Legitimacy Problem for Insurers,” Philosophy & Public Affairs 26, no. 4 (1997): 303–350, 10.1111/j.1088-4963.1997.tb00082.x. 11660435

[13] J. Oh , M. J. Kim , S. Hur , J. Oh , and D.‐S. Kim , “Institutionalizing Health Technology Assessment and Priority Setting in South Korea's Universal Health Coverage Journey,” Health Systems and Reform 9, no. 3 (2023): 2338308, 10.1080/23288604.2024.2338308. 38715186

[14] H.‐Y. Lee , E.‐Y. Bae , K. Lee , M. Kang , and J. Oh , “Public Preferences in Resource Allocation for Insurance Coverage of Dental Implant Service in South Korea: Citizens' Jury,” International Journal of Environmental Research and Public Health 18, no. 8 (2021): 4135. 10.3390/ijerph18084135 PMC8070823 33919799

[15] C. Degeling , S. M. Carter , and L. Rychetnik , “Which Public and Why Deliberate?—a Scoping Review of Public Deliberation in Public Health and Health Policy Research,” Social Science & Medicine (1982) 131 (2015): 114–121, 10.1016/j.socscimed.2015.03.009. 25770463

[16] J. S. Fishkin and R. C. Luskin , “Experimenting With a Democratic Ideal: Deliberative Polling and Public Opinion,” Acta Politica 40, no. 3 (2005): 284–298, 10.1057/palgrave.ap.5500121.

[17] J. S. Fishkin , When the People Speak: Deliberative Democracy and Public Consultation (OUP Oxford, 2009).

[18] R. C. Luskin , J. S. Fishkin , and R. Jowell , “Considered Opinions: Deliberative Polling in Britain,” British Journal of Political Science 32, no. 3 (2002): 455–487.

[19] J. S. Fishkin , Democracy and Deliberation: New Directions for Democratic Reform (Yale University Press, 1991).

[20] A. Aron‐Dine , L. Einav , and A. Finkelstein , “The Rand Health Insurance Experiment, Three Decades Later,” Journal of Economic Perspectives 27, no. 1 (2013): 197–222, 10.1257/jep.27.1.197. PMC3943162 24610973

[21] B. M. Essue and S. Birch , “Blunt Policy Instruments Deliver Blunt Policy Outcomes: Why Cost Sharing Is Not Effective at Controlling Utilization and Improving Health System Efficiency,” Medical Care 54, no. 2 (2016): 107–109, 10.1097/MLR.0000000000000500. 26761725

[22] V. Powell , B. Saloner , and L. M. Sabik , “Cost Sharing in Medicaid: Assumptions, Evidence, and Future Directions,” Medical Care Research and Review 73, no. 4 (2016): 383–409. 10.1177/1077558715617381 PMC4879115 26602175

[23] L. M. Sabik and S. O. Gandhi , “Copayments and Emergency Department Use Among Adult Medicaid Enrollees,” Health Economics 25, no. 5 (2016): 529–542, 10.1002/hec.3164. 25728285

[24] A. Tzeel and J. Brown , “Expect the Unexpected: A Role for Behavioral Economics in Understanding the Impact of Cost‐Sharing on Emergency Department Utilization,” American Health & Drug Benefits 3, no. 4 (2010): 248–256. PMC4106596 25126317

[25] N. Daniels , Just Health: Meeting Health Needs Fairly,” in ed. (Cambridge University Press, 2008).

[26] J. Abelson , P.‐G. Forest , J. Eyles , P. Smith , E. Martin , and F.‐P. Gauvin , “Deliberations About Deliberative Methods: Issues in the Design and Evaluation of Public Participation Processes,” Social Science & Medicine (1982) 57, no. 2 (2003): 239–251, 10.1016/s0277-9536(02)00343-x. 12765705

[27] E. Blacksher , A. Diebel , P. G. Forest , S. D. Goold , and J. Abelson , “What Is Public Deliberation?,” Hastings Center Report 42, no. 2 (2012): 14–16, 10.1002/hast.26. 22733324

[28] M. X. D. Carpini , F. L. Cook , and L. R. Jacobs , “Public Deliberation, Discursive Participation, and Citizen Engagement: A Review of the Empirical Literature,” Annual Review of Political Science 7 (2004): 315–344.

[29] B. Manin , “Political Deliberation & the Adversarial Principle,” Daedalus 146, no. 3 (2017): 39–50.

[30] S. Solomon and J. Abelson , “Why and When Should We Use Public Deliberation?,” Hastings Center Report 42, no. 2 (2012): 17–20, 10.1002/hast.27. PMC3645483 22733325

[31] N. Curato , J. S. Dryzek , S. A. Ercan , C. M. Hendriks , and S. Niemeyer , “Twelve Key Findings in Deliberative Democracy Research,” Daedalus 146, no. 3 (2017): 28–38.

[32] C. Lafont , “Can Democracy Be Deliberative & Participatory? The Democratic Case for Political Uses of Mini‐Publics,” Daedalus 146, no. 3 (2017): 85–105.

[33] C. Lafont , “Deliberation, Participation, and Democratic Legitimacy: Should Deliberative Mini‐Publics Shape Public Policy?,” Journal of Political Philosophy 23, no. 1 (2015): 40–63, 10.1111/jopp.12031.

[34] P. Pettit , “Depoliticizing Democracy,” Ratio Juris 17, no. 1 (2004): 52–65, 10.1111/j.0952-1917.2004.00254.x.

[35] S. Holm , P.‐E. Liss , and O. F. Norheim , “Access to Health Care in the Scandinavian Countries: Ethical Aspects,” Health Care Analysis 7, no. 4 (1999): 321–330, 10.1023/A:1009460010196. 10787795

[36] OECD , Health at a Glance 2023: OECD Indicators (OECD Publishing, 2023).

[37] A. Lupia and A. Norton , “Inequality Is Always in the Room: Language & Power in Deliberative Democracy,” Daedalus 146, no. 3 (2017): 64–76.

